# A Novel p.G141R Mutation in *ILDR1* Leads to Recessive Nonsyndromic Deafness DFNB42 in Two Chinese Han Families

**DOI:** 10.1155/2018/7272308

**Published:** 2018-04-16

**Authors:** Xueling Wang, Longhao Wang, Hu Peng, Tao Yang, Hao Wu

**Affiliations:** ^1^Department of Otorhinolaryngology-Head and Neck Surgery, Shanghai Ninth People's Hospital, Shanghai Jiao Tong University School of Medicine, Shanghai, China; ^2^Ear Institute, Shanghai Jiao Tong University School of Medicine, Shanghai, China; ^3^Shanghai Key Laboratory of Translational Medicine on Ear and Nose Diseases, Shanghai, China; ^4^Department of Otolaryngology-Head and Neck Surgery, Changzheng Hospital, Second Military Medical University, Shanghai, China

## Abstract

Genetic hearing impairment is highly heterogeneous. In this study, targeted next-generation sequencing (NGS) in two Chinese Han families identified a novel p.G141R homozygous mutation in *ILDR1* as the genetic cause of the deafness. Consistent with the recessive inheritance, cosegregation of the p.G141R variant with the hearing loss was confirmed in members of both families by PCR amplification and Sanger sequencing. SNP genotyping analysis suggested that those two families were not closely related. Our study showed that targeted NGS is an effective tool for diagnosis of genetic deafness and that p.G141R in *ILDR1* may be a relatively frequent mutation for DFNB42 in Chinese Hans.

## 1. Introduction

Approximately one in every thousand children suffers congenital or early-onset deafness. In more than half of such cases the hearing impairment can be attributed to genetic causes [1]. To date, more than 100 genes and 150 loci have been identified to be associated with nonsyndromic hearing impairment (Hereditary Hearing Loss Homepage) (http://hereditaryhearingloss.org), with approximately 80% of cases being autosomal recessive [2].

Mutations in the *ILDR1* gene (OMIM 609739) lead to autosomal recessive nonsyndromic deafness DFNB42. *ILDR1* encodes the immunoglobulin-like domain containing receptor 1, a predicted type 1 transmembrane protein. It is widely expressed in a variety of tissues including prostate, testes, pancreas, and kidney. In 2011, Borck et al. first reported several loss-of-function mutations in *ILDR1* resulting in autosomal recessive hearing impairment DFNB42 [3]. *Ildr1* was found expressing in hair cells and supporting cells of the developing mouse cochlea and vestibule [3]. In *Ildr1* knockout mice, hair cells undergo postnatal degeneration. At P35, all knockout mice had profound sensorineural hearing loss associated with a complete loss of outer hair cells and a disorganization of most stereocilia in inner hair cells [4]. Evidence suggested that IDLR1 functions as a water barrier at the tricellular tight junction [5].

To date, only a limited number (less than 20) of *ILDR1* mutations have been reported for DFNB42 in selected ethnic groups in Asia [3, 6–14]. In the present study, we identify a novel missense mutation in *ILDR1* in two Chinese families with DFNB42.

## 2. Materials and Methods

### 2.1. Subjects and Clinical Evaluations

**Figure 1:**
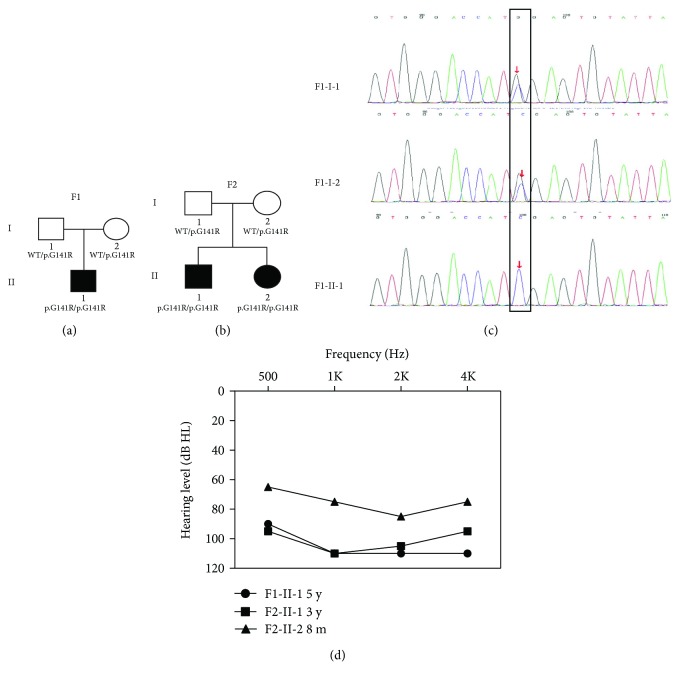
Pedigree and sequencing results of families F1 and F2. (a) Pedigree of family F1. (b) Pedigree of family F2. (c) Representative chromatograms showing the heterozygous (F1-I-1 and F1-I-2) and homozygous (F1-II-1) p.G141R mutation in *ILDR1*. (d) Audiograms of affected members.

This study included two small Chinese recessive deaf families: family 1 (Figure 1(a)) and family 2 (Figure 1(b)). All affected individuals were clinically evaluated in the Department of Otolaryngology-Head and Neck Surgery, Shanghai Ninth People's Hospital, Shanghai, China. The evaluation included a complete medical history interview and a detailed physical examination to rule out possible environmental or syndromic hearing impairment. Auditory evaluations were performed including otoscopic examination, otoacoustic emission (OAE), and pure tone audiometry (PTA). Hearing thresholds of subjects were determined by the air-conduction pure-tone average thresholds ranging from 250 to 8000 Hz. Hearing level was classified as normal (<20 dB), mild (20–40 dB), moderate (41–70 dB), severe (71–90 dB), and profound (>90 dB). Hearing thresholds reported in this study were averages of the right and left ears. Romberg testing and tandem gait were performed for vestibular function examination. Computerized tomography (CT) scan of the temporal bone was carried out for excluding inner-ear anomalies.

### 2.2. Mutation Identification

Informed consent was obtained from all participants or from parents of the young subjects according to a protocol approved by the ethics committee of the Shanghai Ninth People's Hospital, Shanghai Jiao Tong University School of Medicine. Genomic DNA was extracted from peripheral blood samples using a blood DNA kit (TIANGEN Biotech Inc., Beijing, China). Targeted next-generation sequencing was performed in probands of family 1 and family 2 as previously described. Briefly, exon and flanking intron sequences of 159 known deafness genes (Supplementary Table 1) were captured by a customized capture array (NimbleGen, Roche) and sequenced on the Illumina HiSeq2000 analyzer. Data analysis and bioinformatics processing were performed following the standard operation procedure of Illumina. The minor allele frequencies (MAF) of the variants were determined using the gnomAD database (http://gnomad.broadinstitute.org/) and the in-house sequencing data of 200 Chinese Han normal hearing controls. Nonsynonymous variants with an MAF of 0.005 or less and consistent with the autosomal recessive inheritance were considered as candidate pathogenic mutations. Cosegregation of the disease phenotype and the detected variants were confirmed in all family members by PCR amplification and Sanger sequencing. Potential pathogenic effects of the candidate mutations were evaluated by the PolyPhen-2 [15], SIFT [16], and MutationTaster [17] programs.

## 3. Results

### 3.1. Clinical Features

For the three affected individuals in both families (F1-II-1, F2-II-1, and F2-II-2), the hearing impairment was prelingual and bilateral, and the severity ranged from moderate (F1-II-1) to profound (F2-II-1 and F2-II-2) (Figure 1(d)). Speech perception ability was severely impaired in patients F1-II-1 and F2-II-2. No predisposing factors were revealed from their medical and family history. All affected individuals failed the automated auditory brainstem response testing during the neonatal hearing screening and were diagnosed with sensorineural hearing loss. Hearing impairment appeared to be nonsyndromic, and no congenital anomalies, facial dysmorphisms, or intellectual disabilities were reported by the families or detected during physical examination. No vestibular symptoms were recorded.

### 3.2. Identification and Verification of the p.G141R Mutation in *ILDR1*

**Figure 2:**
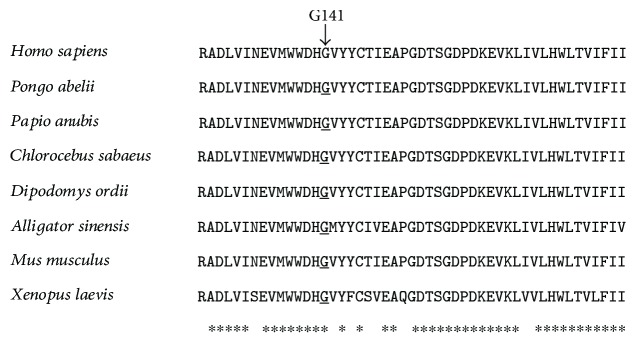
Multispecies sequence alignment of ILDR1 showing the evolutionarily conserved G141 residue.

Targeted next-generation sequencing of 159 known deafness genes in probands F1-II-1 and F2-II-1 identified a total of 8 and 6 variants, respectively (Supplementary Table 2). In both cases, a homozygous c.421G>C (p.G141R) variant in *ILDR1* (NM_001199799) was the only candidate variant consistent with the possible recessive inheritance. Sanger sequencing confirmed cosegregation of this mutation and the hearing impairment in family 1 and family 2, with all affected individuals being homozygous and all parents of the affected being heterozygous (Figure 1). The p.G141R mutation changed codon GGU to GCU in exon 4, substituting an evolutionarily conserved amino acid arginine with histidine in the immunoglobulin superfamily domain of ILDR1 (Figure 2). This mutation was predicted to be pathogenic by programs PolyPhen-2, SIFT, and MutationTaster. It has a minor allele frequency of 0.00009 in gnomAD and was not seen in 200 Chinese Han normal hearing controls. The F1 and F2 families originated from Jiangsu and Zhejiang provinces, respectively, two distinct regions of China. Neither of the families were from consanguineous marriage. Based on sequencing results of *ILDR1* and other deafness genes in chromosome 3, F1-II-1 and F2-II-1 has a distinct genotype for a number of SNPs (Table 1), suggesting that those two probands were not closely related.

## 4. Discussion

In this study, we reported a novel p.G141R mutation in *ILDR1* as the likely genetic cause for the autosomal recessive sensorineural hearing loss (ARSNHL) in two Chinese Han families. Evidence supporting the pathogenic roles of the p.G141R mutation includes: (1) Homozygous p.G141R mutation was identified in all three affected individuals (F1-II-1, F2-II-1, and F2-II-2), and the intrafamilial segregation pattern is consistent with the recessive inheritance (Figure 1). (2) Targeted NGS of 159 known deafness genes identified p.G141R as the only likely pathogenic mutation in probands of both families (Supplementary Table 2). (3) The p.G141R has an extremely low MAF of 0.00009 in 277,128 alleles in the gnomAD database and was absent in 200 ethnically matched normal hearing controls. (4) The p.G141R mutation changes an evolutionarily conserved amino of ILDR1 (Figure 2) and is unanimously predicted to be pathogenic by computational analysis tools PolyPhen-2, SIFT, and MutationTaster. (5) Based on the ACMG guideline [18], the p.G141R variant should be defined as likely pathogenic as it meets with 1 PS4 (the prevalence of the variant in affected individuals is significantly increased compared with the prevalence in controls) and 1 PM2 (extremely low frequency in controls if recessive) criteria.

To date, only 19 *ILDR1* mutations have been reported. Interestingly, most mutations were identified from populations in south and west Asia (Table 2). To our knowledge, our study is the first report of *ILDR1* mutations in Chinese Hans. The two families with the *ILDR1* mutations were identified by targeted NGS of 162 sporadic deaf probands with likely recessive inheritance. The incidence of the pathogenic *ILDR1* variants, therefore, is estimated to be approximately 1.2% in Chinese Han deaf patients. Since homozygous p.G141R mutations were identified in both nonconsanguineous families in the current study and our SNP genotyping analysis suggested that families F1 and F2 were unlikely to be closely related (Table 1), the p.G141R mutation in *ILDR1* may be relatively frequent in Chinese Hans and deserve further screening in expanded studies of deaf patients in China.

**Figure 3:**
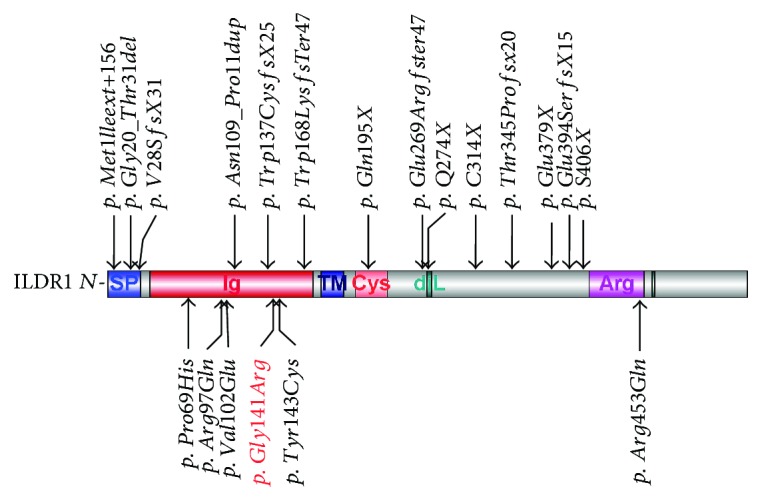
Schematic representation of the functional domains of ILDR1 and the locations of *ILDR1* mutations. The nonmissense and missense mutations are listed above and under the domain structure, respectively.

Of the 19 previously reported *ILDR1* mutations, the majority were nonsense mutations, indels, and stop-codon mutations that significantly truncates or alters the protein structure of ILDR1 (Figure 3, top). Interestingly, 5 of the 6 missense mutations in *ILDR1* including p.G141R were within the extracellular immunoglobulin (Ig) superfamily domain (Figure 3, bottom), suggesting that this domain may have a critical role in relation to the hearing function. On the other hand, the onset and severity of hearing impairment associated with DFNB42 are diverse and there is no clear genotype-phenotype correlation between the missense and nonmissense mutations (Table 2). Our results further verified this point as the affected individuals of families F1 and F2 had moderate and profound hearing impairment, respectively.

Our results also showed that targeted NGS is a powerful tool for the identification of the genetic causes of rare, heterogeneous disorders such as hearing impairment. The implication of this method should be recommended especially when the mutation is rare and the family size is limited, in which cases other methods such as an association study or linkage analysis will not be available.

## 5. Conclusions

The novel p.G141R mutation in *ILDR1* is the likely genetic cause for the hearing impairment in two unrelated Chinese Han DFNB42 families. Targeted NGS is recommended for mutation identification of the rare deafness genes in small families or sporadic cases.

## Figures and Tables

**Table 1:** Genotype of SNPs in chromosome 3 for probands F1-II-1 and F2-II-1.

dbSNP	Chromosome	Position	Gene	Genotype		MAF in Chinese Hans
				F1-II-1	F2-II-1	
rs78962087	3p13	70014199	*MITF*	C/A	C/C	0.0021
rs2306522	3p21.31	45542083	*LARS2*	T/T	T/C	0.1413
rs11549809	3p21.31	45557707	*LARS2*	G/A	G/G	0.1413
rs10578999	3p21.31	46751073	*TMIE*	TAAG/T	T/T	0.7314
p.G141R	3q13.33	121720670	*ILDR1*	G/G	G/G	0
rs2877561	3q13.33	121712051	*ILDR1*	C/C	A/A	0.6304
rs16846663	3q25.1	150658264	*CLRN1*	G/A	G/G	0
rs187218889	3q25.1	150690487	*CLRN1*	G/T	G/G	0.0057
rs3796240	3q25.1	150690566	*CLRN1*	T/T	C/C	0.0630
rs188384	3q28	191074873	*CCDC50*	G/G	C/C	0.6435
rs11542549	3q28	191075902	*CCDC50*	C/T	C/C	0.1652
rs2028573	3q28	191093080	*CCDC50*	A/A	G/G	0.1500
rs4677728	3q28	191093310	*CCDC50*	A/G	A/A	0.0783
rs4677729	3q28	191093384	*CCDC50*	A/A	G/G	0.0152
rs188384	3q28	191094873	*CCDC50*	G/G	C/C	0.6434
rs34031057	3q28	191097928	*CCDC50*	G/G	G/GT	0.0109
rs7624750	3q29	193334991	*OPA1*	G/A	G/G	0.2457
rs166850	3q29	193355074	*OPA1*	C/C	T/T	0.6739
rs10451941	3q29	193355102	*OPA1*	T/C	T/T	0.2000
rs9851685	3q29	193374964	*OPA1*	T/C	T/T	0.2326
rs3772393	3q29	193336639	*OPA1*	T/C	T/T	0.2000

**Table 2:** Summary of mutations in *ILDR1* that are associated with DFNB42.

Mutation (protein)	Affected domains	Hearing phenotype	Ethnic group	Reference
p.Met1Ile*ext*+136	Signal peptide and extracellular domain	Moderate to profound	Pakistan	[3]
p.Gly20_Thr31del	Signal peptide and extracellular domain	Moderate to profound	Iranians	[3]
p.V28SfsX31	Extracellular, transmembrane, and intracellular domains	N/A	Pakistan	[3]
p.Pro69His	Extracellular domain	Postlingual onset and partial deafness	Korean	[11]
p.Arg97Gln	Extracellular domain	N/A	Pakistan	[3]
p.Val102Glu	Extracellular domain	Severe to profound	Iranian	[10]
p.Asn109_Pro111dup	Extracellular domain	Moderate to profound	Saudi Arabian	[9]
p.Trp137CysfsX25	Extracellular domain	N/A	Pakistan	[3]
p.G141R	Extracellular domain	Moderate to profound	Chinese	This study
p.Tyr143Cys	Extracellular domain	Moderate to profound	Iranians	[15]
p.Trp168LysfsTer47	Transmembrane and intracellular domains	Severe	Pakistan	[3]
p.Gln195X	Intracellular domain	Severe to profound	Iranians	[3]
p.Glu269ArgfsTer47	Intracellular domain	Severe to profound	United Arab Emirates	[13]
p.Q274X	Intracellular domain	N/A	Iranian	[8]
p.C314X	Intracellular domain	N/A	Iranian	[7]
p.Thr345ProfsX20	Intracellular domain	Severe	Pakistan	[3]
p.Glu379X	Intracellular domain	Severe to profound	Pakistan	[3]
p.Glu394SerfsX15	Intracellular domain	Severe	Pakistan	[3]
p.S406X	Intracellular domain	Moderate to profound	Iranian	[10]
p.Arg453Gln	Intracellular domain	Severe to profound	Pakistan	[3]

## Supplementary Materials

Supplementary Materials Supplementary Table 1: 159 deafness genes targeted for the next-generation sequencing. Supplementary Table 2: candidate variants identified by targeted NGS for probands F1-II-1 and F2-II-1.
