# Supplementary material for: A Novel p.G141R Mutation in ILDR1 Leads to Recessive Nonsyndromic Deafness DFNB42 in Two Chinese Han Families
Source: Neural Plast. 2018 Apr 16;2018:7272308. doi: 10.1155/2018/7272308 (PMC5926476; doi:10.1155/2018/7272308)
Supplement: Supplementary Materials — Supplementary Table 1: 159 deafness genes targeted for the next-generation sequencing. Supplementary Table 2: candidate variants identified by targeted NGS for probands F1-II-1 and F2-II-1. [file 7272308.f1.docx]

| TYR | USH1C | USH1G | USH2A | WFS1 |  |  |
| --- | --- | --- | --- | --- | --- | --- |

**Supplementary Table 1.** 159 deafness genes targeted for the next-generation sequencing

| deafness related mitochondrial DNA | |
| --- | --- |
| MT-RNR1 | chrM:640-1601 |
| MT-TL1 | chrM:3230-3304 |
| MT-CO1 | chrM:5904-7445 |
| MT-TS1 | chrM:7446-7514 |
| MT-TK | chrM:8295-8364 |
| MT-TE | chrM:14674-14742 |

| deafness related miRNA | |
| --- | --- |
| miR-96 | chr7: 129414532-129414609 |
| miR-182 | chr7: 129410223-129410332 |
| miR-183 | chr7: 129414745-129414854 |

**Supplementary Table 2.** Candidate variants identified by targeted NGS for probands F1-II-1 and F2-II-1

| Probands | Gene | Reference sequence | Variants | Genotype | MAF |  |
| --- | --- | --- | --- | --- | --- | --- |
| F1-II-1  F2-II-1 | TCOF1 | NM_000356 | p.A1313T  (c.G3937A) | het | 0.00005 | |
|  | CDH23 | NM_022124 | p.G3287D  (c.G9860A) | het | 0.00005 | |
|  | ILDR1 | NM_001199799 | p.G141R  (c.421G>C) | hom | 0.00009 | |
|  | MYO7A | NM_000260 | p.C1201S  (c.G3602C) | het | 0.00032 | |
|  | MITF | NM_000248 | p.L354I  (c.C1060A) | het | 0.00217 | |
|  | FREM1 | NM_144966 | p.K1625N  (c.A4875C) | het | 0.00088 | |
|  | TJP2 | NM_004817 | p.L389P  (c.T1166C) | het | 0.000004 | |
|  | USH2A | NM_206933 | p.S4420F  (c.C13259T) | het | 0 | |
|  | CDH23 | NM_022124 | p.D2218E  (c.6654C>A) | het | 0.0011 | |
|  | DSPP | NM_014208 | p.S211N  (c.632G>A) | het | 0.00011 | |
|  | GPR98 | NM_032119 | p.Y6167F  (c.18500A>T) | het | 0 | |
|  | ILDR1 | NM_001199799 | p.G141R  (c.421G>C) | hom | 0.00009 | |
|  | USH2A | NM_206933 | p.S3822P  (c.11464T>C) | het | 0.0014 | |
|  | USH2A | NM_206933 | p.P2210A  (c.6628C>G) | het | 0.00078 | |
